# Unravelling domestication: introduction to the theme issue

**DOI:** 10.1098/rstb.2024.0187

**Published:** 2025-05-15

**Authors:** Rosalind Emma Gillis, Marta Dal Corso, Hugo Rafael Oliveira, Robert N. Spengler

**Affiliations:** ^1^ Referat Naturwissenschaften, Deutsches Archäologisches Institut, 14195 Berlin, Germany; ^2^ ICArEHB, Interdisciplinary Center for Archaeology and Evolution Human Behaviour, University of Algarve, Campus de Gambelas, 8005-139 Faro, Portugal; ^3^ Department of Geosciences, Università degli Studi di Padova, 35131 Padova, Italy; ^4^ Domestication and Anthropogenic Evolution Research Group, Max Planck Institute for Geoanthropology, 07745 Jena, Germany

**Keywords:** domestication, animals, cultivars, feralization, adaptation, landscape development

The domestication of plants and animals is considered one of the key milestones of cultural evolution, on a par with the use of lithic technology and mastery of fire. Domesticated species are—and have been—fundamental to the growth and economic success of human societies. Millennia of herding and agricultural intensification have caused irreversible changes to natural environments, while the ability to accrue and control food surpluses has been linked with the development of complex societies as well as the exacerbation of socioeconomic inequalities. From the mid-Holocene onwards, domesticated plants and animals became integral to the maintenance of human populations and their social orders across a range of contrasting environments. In a few cases, this form of economic production stretches back to the Pleistocene–Holocene transition. The intensification of agricultural systems has led to a series of demographic expansion waves that traversed the globe, and ultimately resulted in the congregation of densely clustered populations [1,2]. The role of plant and animal husbandry in the grand narratives of humanity, especially in relationship to urbanism, the formation of complex, hierarchical political systems and craft or labour segmentation, has been emphasized in scholarship for centuries [3–6].

Many scholars have envisioned a sharp dichotomy between ‘wild’ and ‘domesticated’ both in relation to organisms—crops and animals—and when categorizing subsistence strategies throughout (pre)history. This division can be hard to discern in the archaeological record, and actively downplays the role of wild plant and animal species in human economic and cultural development. As scholars have been pointing out for nearly a century, the bifurcated classification of economies into foraging and farming disregards societies that managed/cultivated wild species, which had not evolved domestication traits and mixed food-procurement strategies [7,8]. The impacts that farming communities have had on global ecosystems have become more pronounced as populations have expanded. The ecological impacts have become more dramatic as agricultural systems have been intensified, driving the evolution of both cultivated and wild organisms in ways that will have ripple effects throughout all future life on Earth [9–11]. Consequently, with humans as the apex ecosystem engineers, this dichotomy between ‘wild’ and ‘domesticated’ is becoming increasingly blurred. Never before in human history has it been so evident that humans are part of nature, they are altering all Earth systems, and are, in turn, acted upon by the biotic and abiotic world. The term 'domesticated' has denoted closeness to humanity, resulting in preferential attention for some organisms in research. In the past, studies have focused on domestication of specific species that were economically important to both modern and ancient communities with [12], more often than not, species being studied in isolation from the ecosystems and mutualisms that they evolved to be part of. This special issue will focus on the origins and impact of domesticated species outside the traditional research spheres, and understudied species, such as opium poppy (*Papaver somniferum* [13]) and reindeer (*Rangifer tarandus*) [14], while at the same time bringing new perspectives on economically important species, such as sheep (*Ovis aries*) and goats (*Capra hircus*) [15], and barley (*Hordeum vulgare*) [16].

Archaeology is well positioned to study the cultural origins of animal husbandry, the development of cultivation and the evolutionary origins of domestication. Supporting this research, the disparate cultures that first engaged in these behaviours serve as ideal comparative case studies, as observed by Dal Corso *et al*. [17]. Farming and herding behaviours were independently converged upon by humans around the world over the course of the Holocene; these practices began in very different cultural arenas and on dramatically different ecological landscapes, from tropical forests to arid deserts and high mountains. No single-lever argument for why or how farming developed can serve this array of scenarios [18]. Despite the differences, there are clear parsimonious models for cultural development drawing on the increased productivity of domesticated organisms and the cultural systems that locked those plants and animals into a mutualism with humanity [19]. For example, by 6500 years ago in northern China a couple of panicoid grasses (broomcorn millet, *Panicum miliaceum,* and foxtail millet, *Setaria italica*) began to evolve in ways that better suited life on an anthropogenic landscape; a few semi-aquatic populations of rice (*Oryza sativa*) started evolving similarly in central China around 6000 years ago [20,21]). Nearly, coeval with these evolutionary changes, humans established villages and remained more stationary throughout the year, as evidenced at the same sites in eastern China where the earliest archaeobotanical remains of domesticated plants were recovered—e.g. Cishan, Dadiwan and Peiligang [20,22]. Isotopic evidence from human bone collagen confirms the link between a greater grain-based diet and the increased density of villages across East Asia by 5000 years ago, at the same time that the demic wave began expanding outward [20,23]. Domesticated animals played an important role in this expansion: in Neolithic northern China, pigs (*Sus scrofa domesticus*) were actively integrated into the cultivation system that has been proposed to be central to the development of complex societies [24]. Meanwhile, in the eastern European forest-steppe, the Trypillia communities of the Chalcolithic relied on cattle (*Bos taurus*) manure for cultivation of pulses and cereals in an integrated economic system [25]. Sedentarism is intricately linked to crop cultivation, whereas seasonally mobile societies are often associated with herding animals, such as sheep, goats or reindeer. Recognizing that the mutualistic relationships between humans and a select handful of plants and animals supported many of the key cultural changes that led humanity into modernity, then a clearer understanding of what domestication is and how it unfolded in prehistory is one of the most important areas of study spanning the social and biological sciences.

Much of the scientific understanding of the process of domestication has been built on genetic and archaeological studies of the most economically important food species, for example, wheats (*Triticum* sp. [26]), rice [27,28], cattle [29], pigs [30,31] and chickens (*Gallus gallus domesticus*) [32,33]. Most studies have also focused on the main centres of domestication, i.e. East and Southwest Asia. The role of less prominent species has been downplayed in narratives about the domestication process, as is the case for many legumes, textile crops or secondary animal species, such as ducks (mallard: *Anas platyrhynchos*; Muscovy duck: *Cairina moschata*) and reindeer. While the studies of core crops and animals have advanced knowledge about the evolution of domestication traits, much remains unknown about: (i) the evolution of crops and animals outside the traditional centres of domestication; (ii) the role of feralization in the domestication process in plant and animal species; and (iii) the long-term impacts of domesticated species on various environments. In order to address these three gaps in the literature, we have brought together a group of leading experts to contribute to this theme issue, titled ‘Unravelling domestication: multi-disciplinary perspectives on human and non-human relationships in the past, present and future’. The papers in this issue aim to tackle these challenges by bringing a fresh perspective on the definition of 'domestication' with case studies and essays concerning understudied animals and plants globally and using multidisciplinary approaches. The issue is split into three interconnected sections: §1 Reviewing the past and defining future directions; §2 New perspectives in the study of domestication; and §3 Domestication within the landscape.

## 1. Reviewing the past, seeking directions

One of the greatest hindrances to scientific advancement is semantics; the inability to communicate across scholarly disciplines has held back research for centuries. An essential next step in the study of domestication is the construction of a generally agreed-upon definition of what domestication is. How can a field of scholarship advance, if the specialists cannot describe what they are studying? There have been many attempts at defining or redefining the word 'domestication', but they have generally fallen short of meeting consensus (see [34,35]). Furthermore, the ways that the word has been used are clearly biasing scholarly interpretation; this is most evident in the active usage—as in ‘ancient people domesticated mice’. In their article, Spengler *et al*. [36] push for a passive usage of the word, as in ‘mice evolved domestication traits in ancient times’. They also draw on decades of debates over the definition of a species in order to construct a definition that relies on fewer assumptions or preconceived ideas about what domestication is. The definition they propose is wide-reaching and includes all organisms that have had their evolutionary trajectories significantly altered by humanity. Their definition would include everything from cats to rats and from feeder birds to body lice; the open-ended aspects demonstrate that, despite only labelling as domesticated a small sliver of the life that has existed on Earth, currently humanity is rapidly driving all life on Earth towards domestication.

Modelling the emergence of domesticated species has been a focus of research for decades; however, models have not considered plant and animal domestication together. Gaucherel & Evin [37] use a possibilistic and discrete-event approach to explore the possible trajectories for the emergence of farming and domesticated species, as well as considering the overexploitation of resources. Their methodological approach takes an EDEN (ecological discrete-event networks) framework that was built with three main elements: variables, rules and constraints. The study focuses on Southwest Asia (i.e. the Near East), and, unlike previous models, they consider both wild/domesticated plants and animals together and their relationship with humans. The authors propose three theoretical models: (i) the emergence of agriculture without overexploitation of resources; (ii) overexploitation of domesticated resources; and (iii) overexploitation of both wild and domestic resources without movement from an external pool of animals. They highlight the complete absence of a synthesis of plant and animal remains across the region. Moreover, the very nature of plant and animal remains has influenced domestication research, and the contrasting reproduction characteristics. In light of their models, they argue for the possibility that animal domestication took place earlier than presently proposed [38], and controversially earlier than cereals.

Fuller *et al*. [39] dive into the understudied topic of weeds, with a focus on weedy plants that evolved to become important agricultural crops. They take the process of commensalism in weeds and break it into two categories of domestication, ruderal and segetal, which they envision as playing out in both similar and contrasting ways. Notably, ruderal pathways to domestication—in these authors' schema—involve weedy plants that adapted to an anthropogenic ecosystem around ancient villages. As examples, they envision cucurbits, such as squash (*Cucurbita* spp.) and melons (e.g. *Cucumis* spp.), as well as chilli (*Capsicum* spp.), evolving traits of commensalism and eventually domestication in this way. Their segetal pathway involves weeds in agricultural fields evolving traits to better adapt to highly disturbed anthropogenic ecosystems. These adaptations set the plants up to become crops. The authors use rye and some of the millets as case studies here. They show that there is only a thin division between plants that end up in the weed pile and those that become economically significant.

## 2. New perspectives on domestication

New approaches, including stable isotopes, genetics, and geometric morphometric analysis, coupled with improved computational power, are allowing big-data analyses of measurements collected over the last 50 years, providing new insights into the domestication process and the emergence of traits. The second section of this theme issue presents new perspectives on common and economically important species, such as sheep, goats, chickens, barley and other cereals, and tree crops. Sheep and goats are part of the cohort of species that were domesticated early in Southwest Asia, and have been of primary importance to farming communities across Eurasia since their domestication. While having similar morphology and similar environmental tolerance, they have different productive capabilities. Geometric morphometric analysis of the third molars of sheep and goats from the northwestern Mediterranean by Jeanjean *et al*. [15] demonstrates that for 8000 years, from the sixth millennium BCE, sheep were under greater selection than goats. While together they have been associated with early Neolithic farmers, they were on different evolutionary tracks. The authors propose that the differences may be due to sheep’s multi-productive capabilities (i.e. meat, milk and wool) in comparison with goats. In a similar vein, barley is one of the most important grasses to evolve mutualistic traits alongside humans; it served as a staple crop across much of Eurasia. In the study by Jeanty *et al*. [16], nearly 10 000 ancient barley grains coming from contexts spanning the Neolithic to the Medieval periods were morphologically analysed. The grains, originating from southern France and Catalonia, illustrate changes in the prevailing morphotypes through time and across space. The researchers have shown contrasting preferences between two-rowed and six-rowed forms, as well as between naked and hulled types. These data show waves of transition as crops were adopted, abandoned and reintroduced cyclically over time.

Seed size increase, one of the earliest domestication traits, is the core of the work by Dal Martello *et al*. [40], who also consider seed morphometrics of some of the main cereal crops in Europe and Asia (barley, free-threshing wheat (*Triticum aestivum*), broomcorn millet and foxtail millet) from a transcontinental and diachronic perspective. The linear measurements that they use allow them to detect parallel or divergent evolutionary trends within lineages of these cereals outside their centres of origin, after dispersal into new environments. Unpublished datasets from Central Asia and China, compared with published evidence, show that increases in seed size occurred initially within the centres of origin at a rate that plateaued after a few millennia. Relatively speaking, domestication occurred rapidly in the beginning and then slowed down. However, size increase never fully stopped, as modern landraces demonstrate, this ability to continue increasing in size shows that the plateau in the rate of change is not a product of a biologically determined absolute constraint.

One of the ways that agriculture was directly embedded with animal husbandry is through the use of animal manure to return nutrients to heavily cultivated soils. Rye has received a great deal of attention recently because of its ability to thrive on marginal lands with poor soils and/or high salinity, and thus it is important for sustainable agriculture. Schlütz *et al*. [41] apply stable isotopic analysis of carbonized archaeological grains to investigate farming practices among past German societies, namely relating to the use of rye. Stable isotopic analysis of plants and animals allows us to directly assess the ecological niche where these species were raised. Manuring was frequently used as a strategy to increase productivity, allowing more intensive cultivation with fewer fallow years. Here, the authors combined the isotopic analysis of rye grains obtained from manuring experiments with that of archaeological grains, providing a glimpse into agricultural strategies and the role these practices had on societal organization. The article pays homage to Karl-Ernst Behre, a pioneer of archaeobotany and palaeoecology, and one of the first archaeologists to study rye’s domestication and spread, whose 90th birthday was celebrated this year.

Russian agronomist Nikolai Vavilov (1887−1943) is credited with having coined the term ‘centre of origin’ to refer to areas around the globe where the diversity of a particular crop (or group of crops) was highest, reasoning that these locations must have been where the crops were first domesticated. One of the proposed centres was Abyssinia, which includes parts of present-day Ethiopia, Eritrea and Somalia. While subsequently disproven, Vavilov proposed that emmer (*Triticum turgidum* ssp. *dicoccum*), barley, oats (*Avena sativa*) and lentils (*Lens culinaris*) could have been independently domesticated there. Nonetheless, new archaeobotanical data suggest that some crops did originate in the highlands of Ethiopia, namely coffee (*Coffea* spp.), enset (*Ensete ventricosum*), t’ef (*Eragrostis tef*) and finger millet (*Eleusine coracana*). However, the domestication process of these crops is difficult to infer owing to: (i) the paucity of archaeological botanical remains; (ii) the occurrence of wild and feral forms there and in other regions of Africa; and (iii) habitat destruction that has led to the disappearance of populations of the putative wild progenitors of these crops. Mekonnen *et al*. [42] address these issues by analysing the genomes of heirloom varieties of two crops, t’ef and finger millet (as well as wild species in their genera), using the same genomics methods. Using a panel that includes African as well as Asian regions where the cultivation of these crops later spread, they were able to confirm the nature of the wild progenitors and propose routes of diffusion. This work is part of an effort to investigate the emergence of agriculture outside the core areas (e.g. Southwest Asia, Mesoamerica) and to work minor crops into the narratives of plant domestication. Moreover, the genetic data generated can be used for plant breeding efforts by African scientists and institutions; for example, accessions genotyped can be tested for yield and for stress resistance, while publicly available genomic data could be used for allele mining and marker-trait association studies, accelerating the crossing of high-interest varieties. These examples illustrate the potential social and economic impacts of domestication research using archaeobotanical and modern data.

The importance of archaeological material is evident in the study of domestication in the past, while integrating studies of modern plants and animals as well as traditional farming communities is essential for understanding trait selection. The phenotypic and behavioural changes brought about by human action that distinguish domesticated plants and animals from their wild progenitors are known as the ‘domestication syndrome’. Research efforts have been devoted to identifying these domestication traits and their genetic basis [43,44]. Wright and Jensen have defined the term ‘domestication syndrome’, conducting research on how domestication affected species, such as dogs and chickens [45,46]. In this theme issue, Wright *et al*. [47] consider the effect of domestication on the vocalizations of red junglefowl and the genetic basis of one of the most idiosyncratic traits of chicken: the crowing of the male. They combine spectrograms of wild and domesticated birds with the genetic analysis of the progeny of crosses between wild red junglefowl and White Leghorn chicken breeds. These results demonstrate the genetic basis behind subtle differences in the vocalizations of the domesticated chicken, a trait that is silent in the archaeological record.

Preservation and taphonomic processes tend to distort ancient plant remains. Consequently, by investigating modern plants it is possible to determine functional trait selection. Olive trees form a central part of Mediterranean landscapes and economies, providing important sources of oil, while olive groves also support rich biodiversity. Using a trait-based analysis approach of modern Moroccan olive varieties, Kassout *et al*. [48] discuss the functional responses of plant species at the intraspecific level along the continuum of wild–domesticated. Their analysis reveals a pattern of variability that enhances our understanding of the domestication of olive trees, in particular the continued use of wild trees after domestication. Functional traits, such as leaf area and leaf dry matter content, are influenced by regional and local scales, while stomata density is important for adaptation to low water availability—traits that are invisible in the archaeological record. Some of these traits can be passed on through grafting with wild species, similar to traditional practices, allowing the enhancement and development of varieties resilient to stressful conditions. Traditional practices could play an important role in developing species that are resilient to ongoing climate change.

Finally, multi-analytical perspectives that integrate both archaeological and modern material provide a holistic view of domestication processes. The work by Machado *et al*. [13] focuses on the domestication of the opium poppy, explored through genomics and seed morphometrics. Based on comprehensive data collection, the authors trace the history of this crop in Europe and Southwest Asia. The analysis demonstrated that the only taxon genetically close to *Papaver somniferum* is *Papaver setigerum*, autochthonous to the western Mediterranean. This contradicts an alternative hypothesis about the introduction of the crop in Europe as a weed within the Neolithic package from Southwest Asia. Furthermore, differences in seed size and form delineate different evolutionary pressures, suggesting a recent selection for varieties rich in opiates, as opposed to varieties used for poppy seed production. Moreover, this work shows the occurrence of feral forms and introgression events.

## 3. Domestication within the landscape

It is important to emphasize the role of the overall ecosystem within the domestication process, and the interconnected relationships between human and non-human species. Windle *et al*. [14] integrate ethnographic data with stable isotopic analysis to examine multi-species relationships in contrasting reindeer herding strategies in boreal Northeast Asia. These animals have been overlooked by researchers and often considered ‘semi-domesticated’—although this, as the authors state, fails to capture the close relationship that herders have with these species. This ‘semi-domesticated’ status may be a reflection of the continuous use of wild reindeer for interbreeding with domesticated herds. Windle *et al*.'s study demonstrates that stable isotopes can illustrate complex multi-species communities. In particular, the role of supplementary fodders, here the use of fish, underlines the herders’ ecological knowledge and strengthens the relationship between the humans and their herds. Central to this study is the relationship between domestication processes and the landscape.

Besides single or multi-species insights into domestication processes, a broader, ecosystemic, view about landscape development since early farming is depicted in the review paper by Dal Corso *et al*. [17]. Different lines of evidence are discussed for specific regions in Europe, from the Iberian Peninsula to eastern Europe, where agro-sylvo-pastoral landscapes are recognized today as biodiversity hotspots related to traditional farming practices within regional geophysical features. The paper sheds light on the evidence for early environmental impact by herding practices, as described through multi-proxy, as well as single records, from a long-term perspective that considers the period from the Neolithic to the Iron Age.

Leaving Europe and crossing the Mediterranean, the Sahara has been an important and dynamic region for plant–animal–human dynamics for millennia. Florenzano *et al*. [49] review literature on the archaeobotany and palaeoenvironment of the region, looking at contexts rich in dung as well as biomolecular archives, such as dental calculus, all dating as far back as 10 000 BP. Their review indicates that the majority of plant species used during the Holocene were wild plants, and that may have been used as both human and animal food. These plants may have been moved across regions. Moreover, domesticated varieties, such as pearl millet (*Pennisetum glaucum*), were potentially selected because of their drought resistance, coinciding with increase of aridity after *ca* 4500 cal BP spreading from Mali to West Africa. Plant and tree exploitation and other human activity, such as animal husbandry, influenced the flora composition of the region and the landscape during the Middle and Late Holocene alongside climate change [50].

Domesticated species did not evolve the traits of domestication in a vacuum, rather they were embedded within specific landscapes and ecosystems. Eastern North America is marked by rich mosaics of landscapes that include forests, floodplains and prairies. These ecozones were used and managed by indigenous communities from 7000 BP onwards. Mueller [51] provides a detailed review of the crop choices tied to the Eastern Agricultural Complex (EAC) and the biology of these plants, as well as models for domestication of species, such as goosefoot (*Chenopodium berlandieri*). Based on new research contrasted with scholarship relating to the EAC going back nearly six decades, she builds a picture of the intricate relationship between human societies and the landscapes they inhabit. The review underlines that initially EAC communities never focused on a single crop nor was seed crop cultivation tied to increased sedentism. Changes in subsistence strategies took place when increased flooding led to communities moving from the floodplains, connected to changes in sociocultural dynamics and sedentary behaviour while retaining a diverse subsistence economy based on a number of cultivars. These ways of life were cut short with the arrival of maize, leading, in many areas, to a dominance of that crop. Traditional systems were further lost with the arrival of Europeans. Mueller argues for taking a more historical ecological perspective to future studies that places the landscape at the centre of investigations and ensures knowledge transfer between local indigenous communities and research institutions.

Government policies play an important role in crop choice and dynamics, which can be detrimental to traditional crop species and cause a loss of genetic diversity besides having an impact on people's food availability and nutrition [52]. The ethnographic and archaeobotanical approach given in Filatova’s paper [53] concerns change in crop spectra in the highlands of Odisha, India. In her work, the dynamics leading to crop selection behind present day rice and millet cultivation and consumption are investigated, and its relationship with socio-political, cultural, economic and environmental aspects. In order to investigate farming traditions from a long-term perspective, results are compared with archaeobotanical datasets. This study shows that governmental and international policies have had a considerable impact on highland crop cultivation in Odisha, leading to a decrease in the traditional cultivation and consumption of millets, and an increase in the presence of modern introduced rice varieties, with cultural and economic consequences.

## 4. Conclusions

The study of domestication generates strong emotions among scholars; these responses can turn to lively debate when we approach the question of intentionality. Over the past decades, scholars have contemplated the rates at which ancient domestication traits evolved and how aware of the process ancient farmers were. As domestication scholarship focuses on the questions of why humans domesticated plants and developed cultivation systems, the assumption of intentionality has been so deeply intertwined with archaeological scholarship that many researchers have trouble contemplating other perspectives. Often archaeologists have difficulty separating intentionality in the domestication process from judgments about intelligence. We argue that the only way forward for such research is through comparative approaches, looking across a wide array of species, ecological settings, cultural affiliations and time ranges. This theme issue presents new perspectives, drawing underrepresented crops and animals into the debates, and looks for alternative comparative case studies; in doing so, the scholars represented here feed into debates about what ancient domestication looked like and what the role of prehistoric or early historic farmers was in this process. Hopefully, these expanded case studies will make the possibility of unconscious or unintentional and protracted models of domestication more palatable to specialists.

The papers produced here review the state of the art and collectively propose new directions. The collection of articles in this issue focuses on a wide range of species, from different analytical perspectives using both modern and archaeological material. They highlight the advances made over the last 20 years as well as emphasize the gaps that remain in our knowledge. Multidisciplinary studies can harness the power of analytical approaches, such as genetics, stable isotopic and morphometric analyses, and pulling from both modern and archaeological material. This issue marks a new phase in the investigation of domestication, with a greater focus on underrepresented species, and explores domestication processes beyond the traditional scenarios. Moving away from a singular dominant scientific standpoint, to multi-stranded analysis, will provide researchers with new perspectives on domestication processes and the potential domino effect these processes can have within plant and animal communities.

It is also essential that scholars integrate these observations within a more nuanced look at the local ecological setting under which domestication traits evolved. In the final section of the issue, the authors demonstrate that the external ecological, cultural and political environment plays an important role in the domestication process and the adoption of domesticated species. Through greater consideration of these factors, future scientists will differentiate between intentional and non-intentional trait selection by humans. These will generate novel ideas regarding what domestication is and how it played out in the past. By taking a holistic and multidisciplinary approach and reevaluating previously understudied regions and species, the field can move into exciting new directions.

## Data Availability

This article has no additional data.
